# The First Three Months of COVID-19: Epidemiological Evidence for Two SARS-CoV-2 Strains Spreading and Implications for Prevention Strategies

**DOI:** 10.7759/cureus.29146

**Published:** 2022-09-14

**Authors:** Knut M Wittkowski

**Affiliations:** 1 Research and Development, Advanced Statistics for Drug Exploration, Repurposing, and Approval (ASDERA) LLC, New York, USA

**Keywords:** vulnerable population, lockdown policies, window of opportunity, influenza-like illness, covid-19 deaths, d614g mutation, sir model, mitigation strategies, coronavirus disease (covid-19)

## Abstract

About a month after the COVID-19 epidemic peaked in Mainland China and severe acute respiratory syndrome coronavirus 2 (SARS-CoV-2) migrated to Europe and then the USA, the epidemiological data began to provide important insights into the risks associated with the disease and the effectiveness of intervention strategies such as travel restrictions and lockdowns (“social distancing”). Respiratory diseases, including the 2003 severe acute respiratory syndrome (SARS) epidemic, remain only about two months in any given population, although peak incidence and lethality can vary. The epidemiological data suggested that at least two strains of SARS-CoV-2 had evolved during the first months of the epidemic while the virus migrated from Mainland China to Europe. South Korea (SK), Iran, Italy (IT), and Italy’s neighbors were then hit by the more dangerous “SKII” variant. While the first epidemic in continental Asia was about to end and in Europe about to level off, the more recent epidemic in the younger US population was still increasing, albeit not exponentially anymore.

The same models that help us to understand the epidemic also help us to choose prevention strategies. The containment of high-risk people, such as the elderly with comorbidities, and reducing disease severity, by either vaccination, reduction of comorbidities (seen as risk factors already in Italy), or early treatment of complications, are the best strategies against a respiratory virus disease (RVD). Lockdowns can be effective during the month following the peak incidence of infections when the exponential increase of cases ends (the window of opportunity). From the standard susceptible-infectious-resistant (SIR) model used, containing low-risk people too early, instead, merely prolongs the time the virus needs to circulate until the incidence is high enough to reach “herd immunity.” Containing low-risk people too late is also not helpful, unless to prevent a rebound if containment started too early.

## Introduction

Prologue

The Abstract and the main body (from Introduction to Part I of Conclusions) have been available as a prepublication on medRxiv/bioRxiv from 2020-03-31 (V1), with 2020-04-07 (V2), 2020-04-15 (V3), 2020-04-20 (V4), and 2020-04-29 (V5) updates [1], and some data were added until 2020-05-05 (ISO 8601 date format is used throughout), while several attempts for publication failed, mainly because reviewers and/or editors were concerned about two types of conclusions: 1) conclusions about the emergence of novel virus strains based on empirical data before large databases of sequencing data had been collected and 2) conclusions about the effectiveness and risks of interventions based on other epidemiological models than those that led to the lockdowns starting in late 2020-03 [2], which had been made available on the same prepublication server. To start a discussion about using epidemiological data and models earlier in an epidemic, relevant parts of this publication are still written from the early 2020 perspective with only minor changes and clarifications based on input from reviewers and editors.

Introduction (as of 2020-04)

The first cases of a new coronavirus strain, termed severe acute respiratory syndrome coronavirus 2 (SARS-CoV-2) by the International Committee on Taxonomy of Viruses (ICTV), were reported on 2019-12-31 in Wuhan, the capital of the Hubei province of China. As of 2020-05-05 3,544,222 symptomatic cases and 250,977 deaths have been reported from virtually every country in the Northern Hemisphere (see section Data). The disease was termed COVID-19 by the WHO on 2020-02-11 and categorized as a pandemic on 2020-03-12, yet the details of the spread and their implications for prevention have not been discussed in sufficient detail.

The virus moved from China via Europe to the USA. The first case in the USA was diagnosed on 2020-02-28 in a long-term skilled nursing facility in Washington State. For most of the first three months of the epidemic, much of the response was driven by fear, stigma, or discrimination, including naming SARS-CoV-2 the “China virus” despite the fact that seasonal respiratory zoonotic pathogens typically originate in China, where live-animal markets provide chances for animal viruses to transmit to humans. The 2020-03-23 Veterans Health Administration’s COVID-19 Response Plan envisioned a pandemic that would “last 18 months or longer and could include multiple waves of illness” (see Wittkowski [1] for reference).

Between 2020-02-14 and 2020-03-16, the US Dow Jones Index fell by 31% from 29,440 to 20,188, raising fears for the economy, in general, and retirement savings, in particular. By the end of 2020-03, the Dow was down at 19,173 (35%) from 2020-02-14. On 2020-03-26, the US Senate approved a $2 trillion stimulus package. Between 2020-03-21 and 2020-04-23, 26.5 million people filed claims for unemployment benefits, representing 16.2% of the labor force [3].

After three months, the epidemic peaked in Asia, Europe, Oceania, and the Americas, and sufficient data became available to discuss important epidemiological characteristics of COVID-19 and the potential impact of interventions. Changes in number of deaths followed the changes in number of cases (albeit at a lower level) by about 1-2 weeks. During these seasons, pandemic cases seemed to have peaked in early April and deaths in mid-April.

As the 2019/2020 seasonal flu season ends in the Northern Hemisphere (unless there should be an outbreak in maritime Asia, India, or Russia), we can explore the infectivity and lethality of the virus (two important characteristics for public health impact of the disease) and the implication for various prevention strategies. An important consideration is whether the interventions in several countries started too early (prolonging the time the virus stayed in the population and potentially increasing the number of deaths and waves) or too late (being ineffective) and whether there is an optimal time to make the most meaningful impact on the spread of a virus, a crucial time point when a public health intervention should start during the course of the epidemic. In this paper, the data available from the first three months of the epidemic are used to compare responses and outcomes in various countries mainly in the Northern Hemisphere.

## Materials and methods

Data (as of 2020-04)

**Figure 1:**
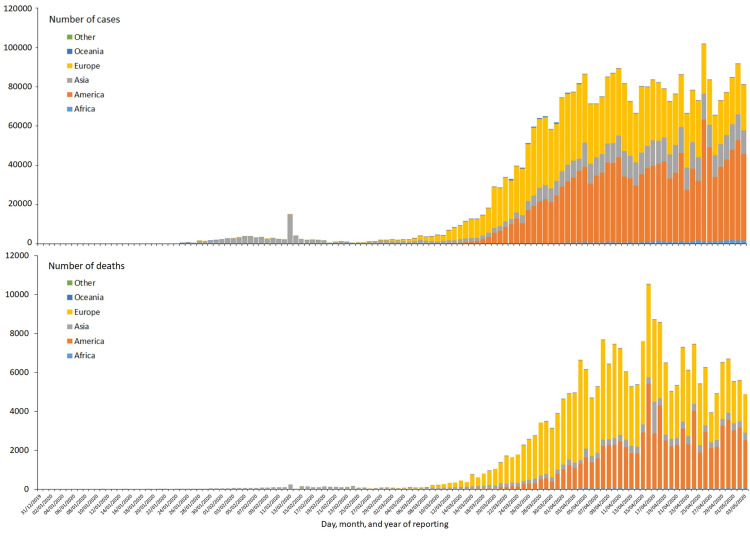
New case reports (top) and death reports (bottom) by continent. Cases (top) and deaths (bottom) were reported in accordance with the applied case definition and testing strategies in the affected countries. Starting with China (Hubei province) on 2020-02-13, the case definitions have been expanded frequently in various countries. The graphs were accessed on 2020-05-04 on the parts of the European Centre for Disease Prevention and Control (ECDC) website (now defunct).

Data of cases (Figure 1, top) and deaths (Figure 1, bottom) were downloaded on 2020-05-05 from the European Centre for Disease Prevention and Control (ECDC) website [4], updates from the Johns Hopkins online tracker [5], and New York City (NYC) data from the official website of the City of New York [6].

**Figure 2:**
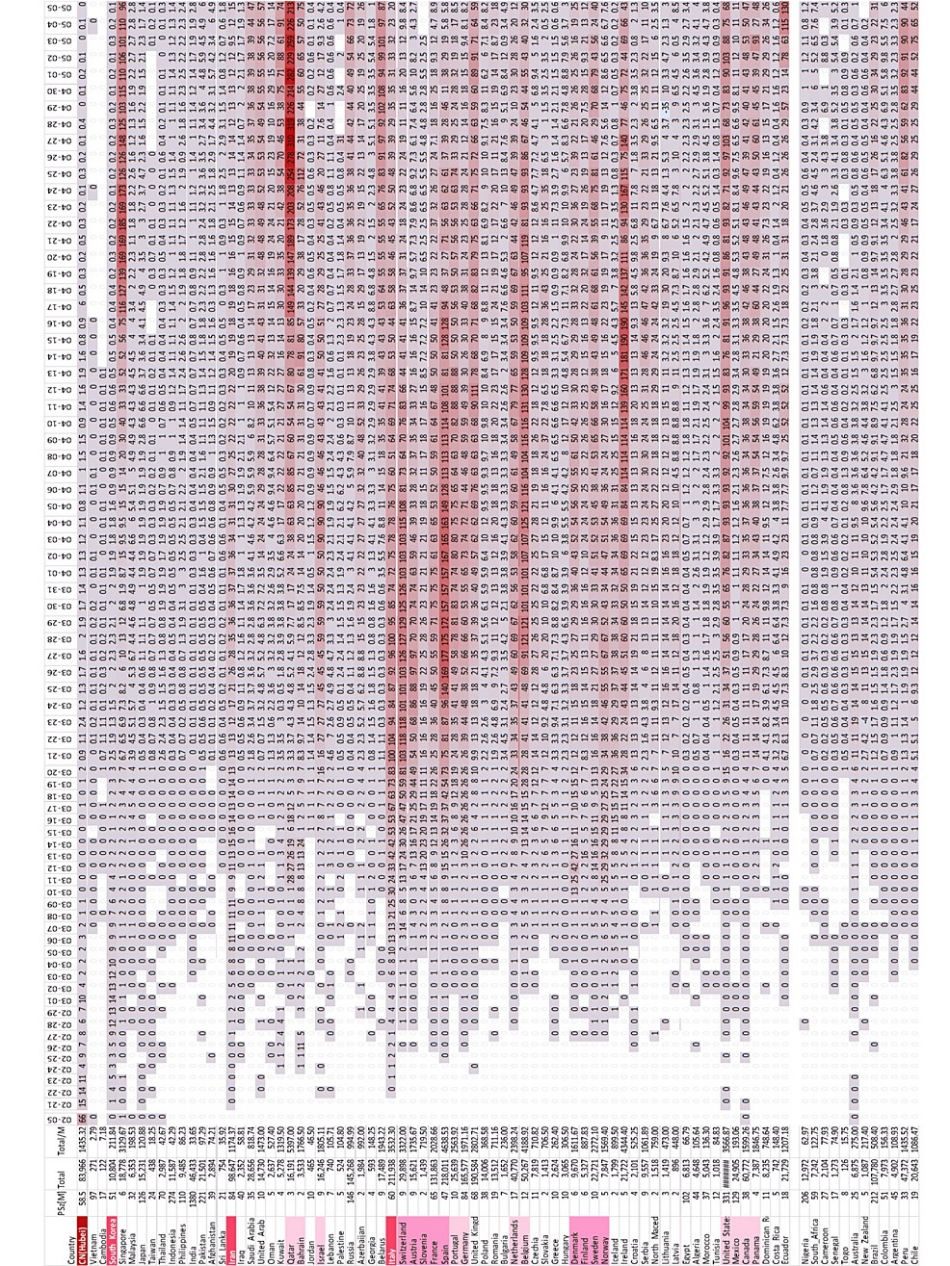
Case incidence by country. Dates: 02-05 (peak intensity in Mainland China, mostly Hubei and neighboring provinces), 2020-02-19 to 2020-03-22. Countries with low population size (PSz) or a low number of cases (total) are hidden. Red background indicates countries/dates with high incidence per capita. Countries/regions are grouped by proximity to each other and distance from Mainland China. Countries with high incidence are indicated by red background.

Figure 2 shows the raw daily incidence by population sizes for countries with epidemiological relevance in the Northern Hemisphere. Countries within proximity are grouped by their peak incidence (red background). It should be noted, however, that definitions of “cases” differ. In some countries/time points, a “case” needs to have symptoms; in others, “cases” can be asymptomatic or only have antibodies (be immune).

Statistical and bioinformatics methods

The data were processed with Microsoft Excel (Microsoft® Corp., Redmond, WA). To avoid biases from inappropriate model assumptions, only basic descriptive statistics were employed. In some cases, data from only the day before or after (or both) were averaged (up to a three-day moving average) to reduce the effects of apparent reporting artifacts (Darwin’s natura non facit saltum) without creating undue biases. The three “smoothers” applied were 1) averaging x_0_ with a previous x_−1_ (or, rarely, following x_+1_) data, 2) applying a moving average of (x_−1_, x_0_, x_+1_) → (2 x_−1_ + x_0_, x_−1_ + x_0_ + x_+1_, x_0_ + 2 x_+1_)/3, and 3) moving averages of the four days leading up to the current date to guide with visual impression. No other changes were applied to the data.

Like China in mid-February, the German Robert Koch Institute (RKI) changed the reporting system in mid-March, which resulted in a near sixfold increase of the data reported on 2020-03-20 over the data reported on 2020-03-19. To account for such isolated event, the data of this and several previous days were averaged (as indicated in the figures) under the assumption that those changes reflect cases that had previously been diagnosed but were not reported.

Epidemiological models

**Figure 3:**
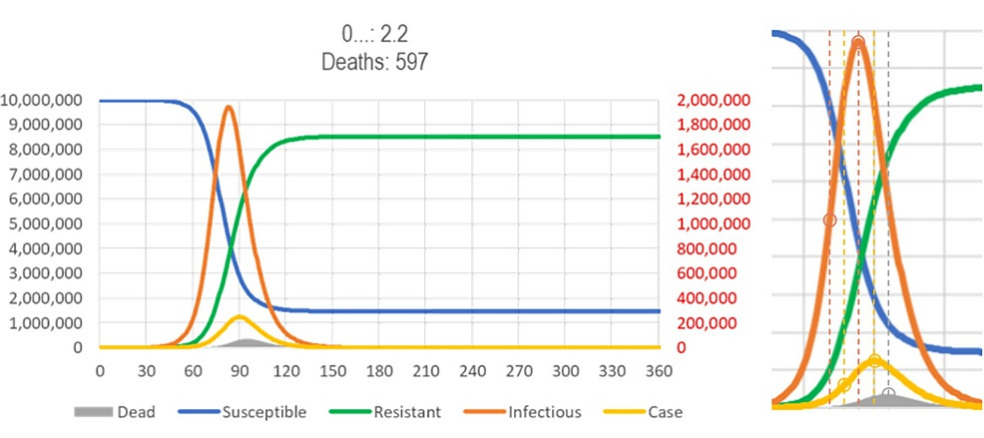
SIR model of SARS. The number of susceptible (blue, left scale), infectious (red, right scale), resistant (green, left scale), cases (orange, arbitrary scale), and dead (gray, arbitrary scale) people after a population of 10,000,000 susceptible people is exposed to 20 subjects carrying a novel virus. Assumptions: reproduction number (R0) = 2.2 and infectious period = seven days (available from https://app.box.com/s/pa446z1csxcvfksgi13oohjm3bjg86ql). SIR: susceptible-infectious-resistant; SARS: severe acute respiratory syndrome.

If a disease causes immunity after an infectious period of a few days only, such as respiratory virus diseases (RVDs), an epidemic ends by itself as the proportion of immune people increases. Under the susceptible-infectious-resistant (SIR) model [7,8], for a reproduction number (R0) (secondary infections by direct contact in a susceptible population) of 1.5-2.5 over seven days (recovery rate: γ = 1/7 = 0.14), the noticeable part of the epidemic lasts about 90-45 days (R0/γ = β = 0.21-0.36) in a homogeneous population of 10 million. The period is shorter for smaller, more homogenous populations and longer for larger, more heterogenous populations. For a given infectious period of 1/γ (here, e.g., seven-day COVID-19 incubation period plus two days), R0 also determines how long it will take for early cases to become visible after a single import (150-160 days), the peak prevalence of infections (5%-22%), and how many people will become immune (55%-90%). To allow for comparisons between models, an arbitrary proportion of symptomatic cases among those becoming infected (0.05%) is used, and 2% of cases are assumed to die (Figure 3).

The model reflects that the key milestones of the epidemic, the inflection point/half maximal point in infections (day 68, red), the inflection point/half maximal point in the number of cases (day 75, orange), the peak in infections (day 83, red), the peak in cases (day 70, orange), and the peak in deaths (day 77, gray), follow each other, following the previous milestone after about a week.

## Results

Time course by country/region (as of early 2020)

Northern Hemisphere

**Figure 4:**
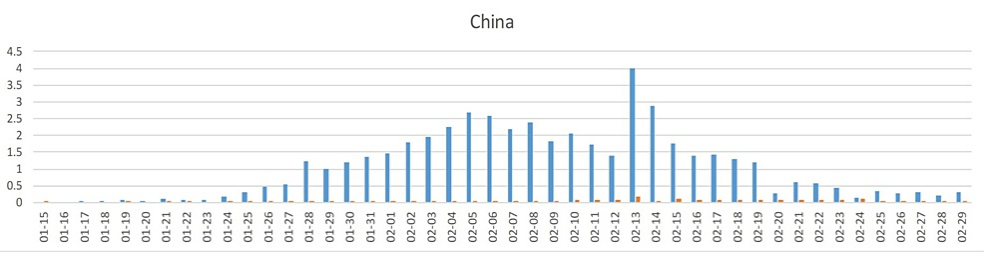
COVID-19 cases in Mainland China. Blue: cases/million/day; red: deaths/million/day. In around 2020-02-13, the case definition was expanded, resulting in additional cases from previous days being added. Hence, the 2020-02-13 cases have been truncated. Most cases were seen in the Hubei province of 58.5 million people (see Figure 2 for population sizes).

Among the Hubei population of 58.5 million, the incidence rose from the first case reported in late 2019 to about 60 new cases per million people per day by 2020-02-05 and then steadily declined (Figure 3) from ~4000 on 2020-02-05 to below 50 cases per day since 2020-03-08. Since 2020-03-31, no cases were reported in Vietnam and only isolated cases in Cambodia (Figure 2). The cumulative incidence was about 1,400/million (Figure 4).

**Figure 5:**
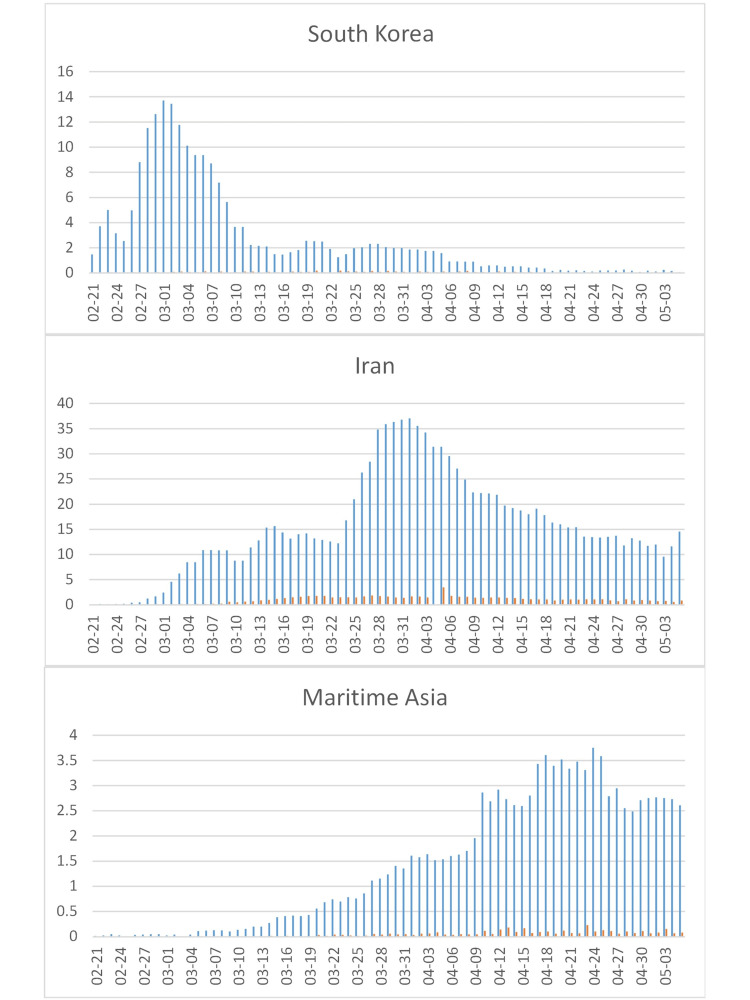
COVID-19 cases in Asia. See Figure 4 for legend.

In continental South Korea (SK) (population: 51 million; cumulative incidence: 210/million), the incidence rose to a peak of about 14/million/day between 2020-02-29 and 2020-03-02, before declining to continuing low rate of less than 150 cases per day (~2/million/day) since 2020-03-12 (Figure 5, top). In Iran (cumulative incidence: 1,174/million), incidence began to rise about a week after South Korea. The top incidence before a new wave started on 2020-03-23 (~15.5 cases/million/day) was about the same. The increase thereafter with a peak around 2020-04-01 at 37 million/day may indicate a “rebound” into a population not sufficiently immunized by the previous wave(s). Lethality in Iran was notably higher than in South Korea and followed the increase in cases (see the SIR model above) with a delay of several days (Figure 5, middle).

By mid 2020-01, the first cases of COVID-19 were seen in other Asian countries, but incidence remained below about three/million/day outside of continental China (Figure 5, bottom); the incidence rose above five/million/day only in Malaysia/Brunei and Singapore; in Singapore, incidence has increased from 185/million/day on 2020-04-22 to a total of 3,130/million/day. Japan, with the largest proportion of people >65 years of age in the world (28%), remained below ~1/million/day until recently, when incidence increased to ~5/million/day around 2020-04-18 but then declined again.

From 2020-03-19 to 2020-03-20, several European countries have seen a more than twofold increase in the number of cases reported (Germany: 570%, San Marino: 340%, Ireland: 260%, Switzerland (CH): 240%, and Austria: 202%). As natura non facit saltum (Darwin: nature doesn’t jump), such abrupt increases must be, at least in part, the result of reporting or other artifacts. In Germany, for instance, the reporting system was changed between 2020-03-16 and 2020-03-19 so that the number likely includes cases previously reported only through a parallel system. France (F), Italy (IT), and Spain (ES) also reported an unusual increase by 27%-35%. Since 2020-03-26, all these countries reported the incidence to decline with a wave pattern consistent with weekday effects of reporting.

**Figure 6:**
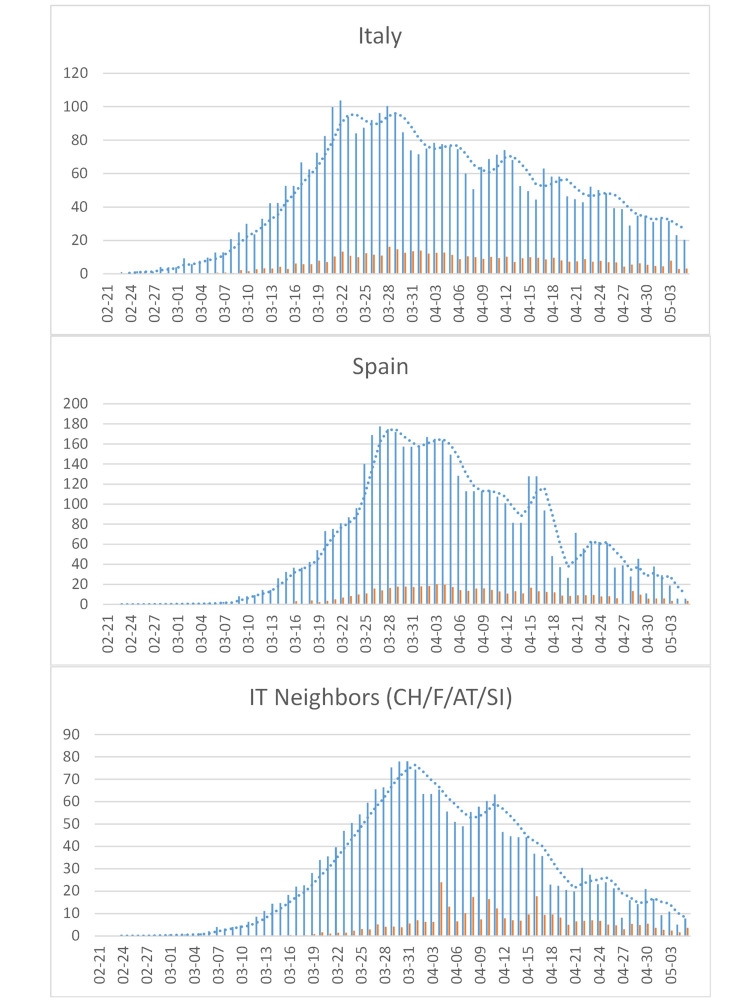
COVID-19 cases in Italy (IT) and its neighboring countries. Italy (top), Spain (middle), and European countries neighboring Italy (bottom). See Figure 4 for legend. CH: Switzerland; F: France; AT: Austria; SI: Slovenia.

**Figure 7:**
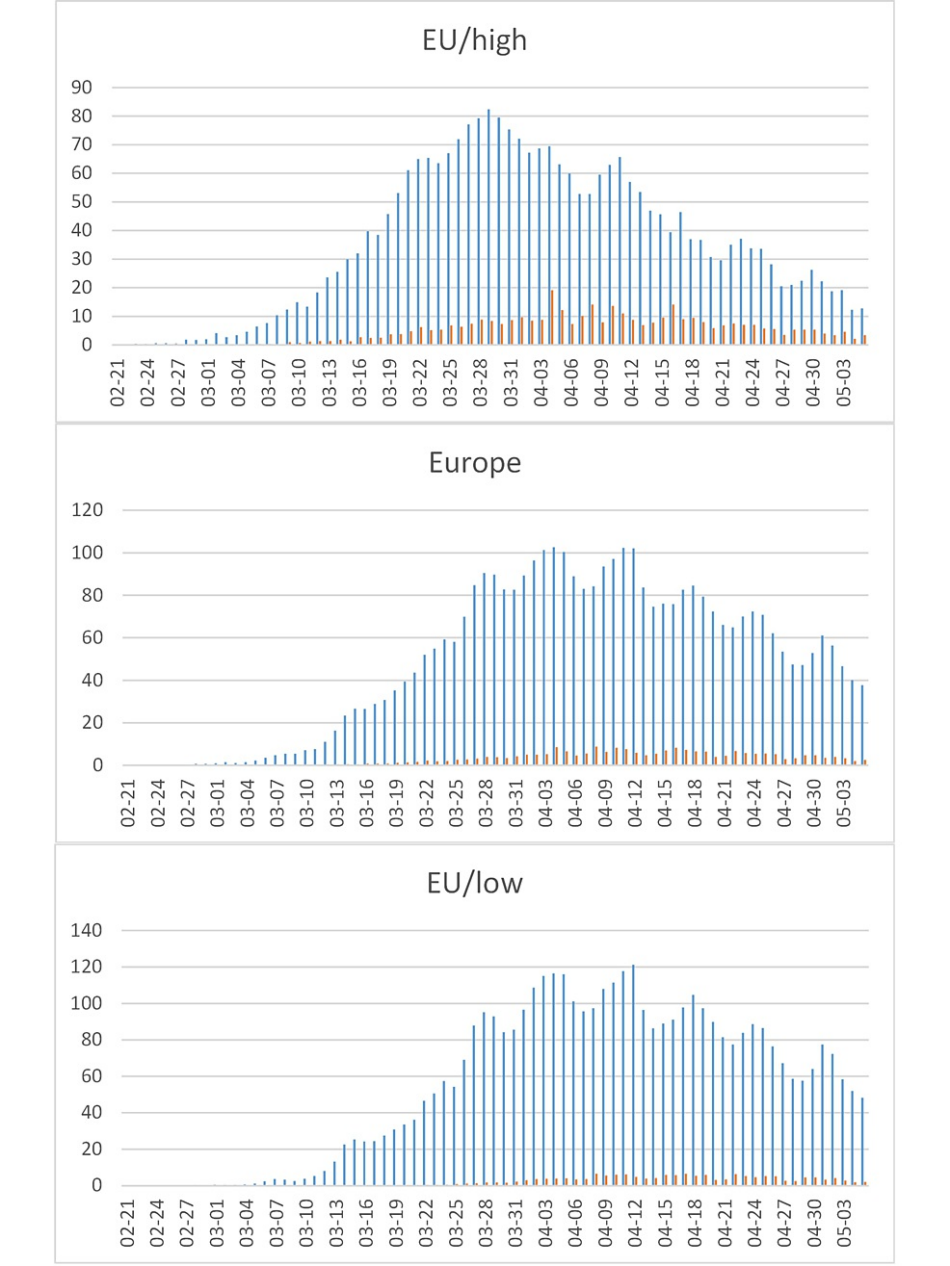
COVID-19 cases in Europe. Early onset/high lethality (Italy {IT} and neighbors, top), total (middle), and late onset/low lethality (other European countries, bottom).  See Figure 4 for legend. EU: European Union.

Cumulative incidence per capita in IT’s neighbors ES and CH exceeded that of IT and Hubei (all but CH with a 47-60 million population). In the European Union (EU), only the Benelux region reached similar levels (Figure 2). In IT (Figure 6) and most EU countries (Figure 7), incidence has been declining slowly from the 100/million/day peak around 03-22, after rising for about four weeks (from 2020-02-26 to 2020-03-22). In Hubei and SK, instead, incidence peaked after only two weeks (Figure 4 and Figure 5) before declining sharply.

**Figure 8:**
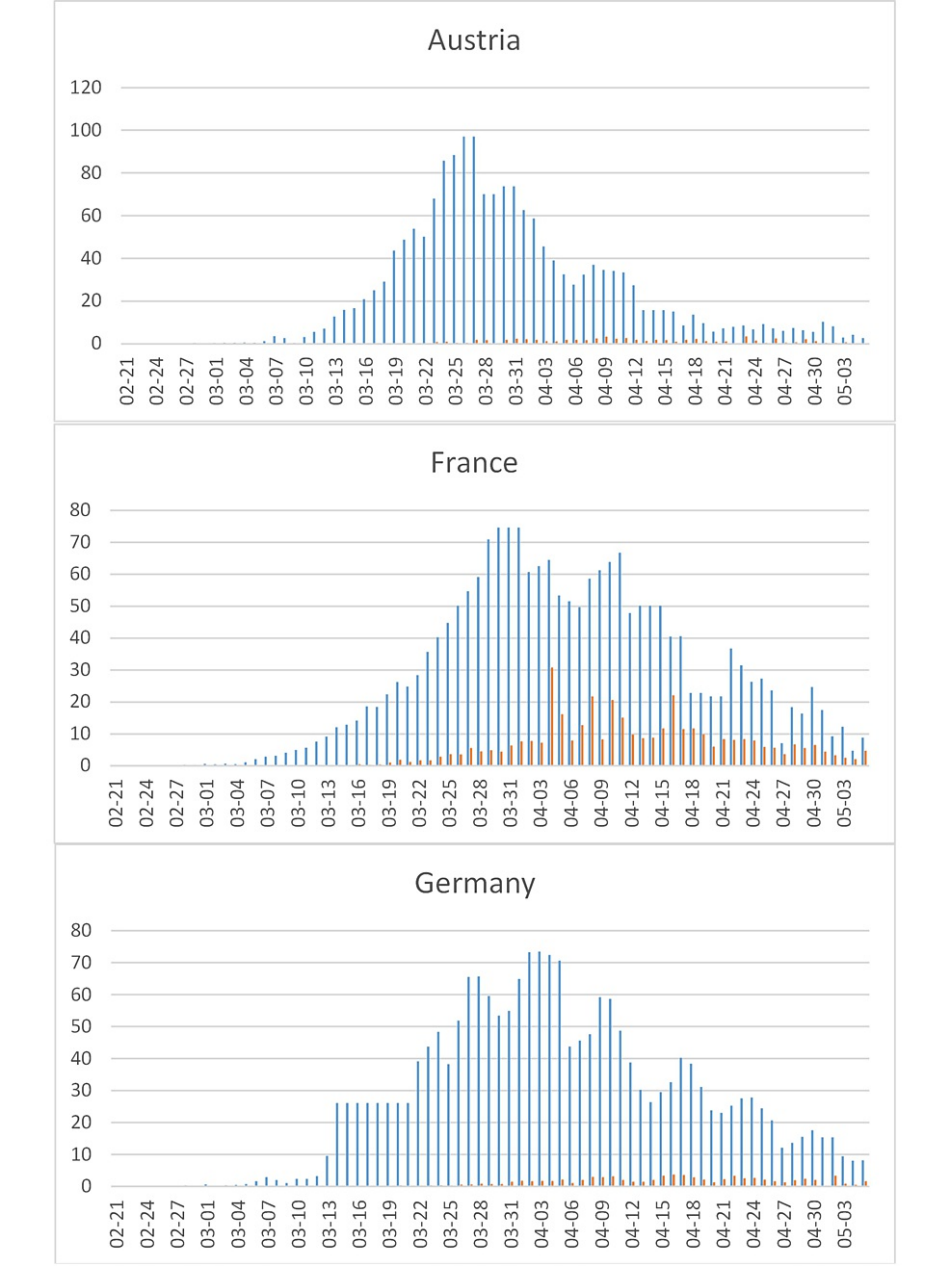
COVID-19 cases in selected European countries. See Figure 4 for legend. Data in Germany are based on cases reported electronically to the Robert Koch Institute (RKI) and transmitted to the Centre for Disease Prevention and Control (ECDC), but the RKI also provides two sources of data on its website that are difficult to reconcile with these data. To account for a temporary peak in Germany on 2020-03-20/21, when the reporting system was changed, the 2020-03-14-21 data were averaged. Six thousand eighty-two cases were added for Germany based on updated data.

Austria (Figure 8) is notable for both its early peak and steep decline; since infections predate cases by at least a week, SARS-CoV-2 in Austria may already have been eradicated.

**Figure 9:**
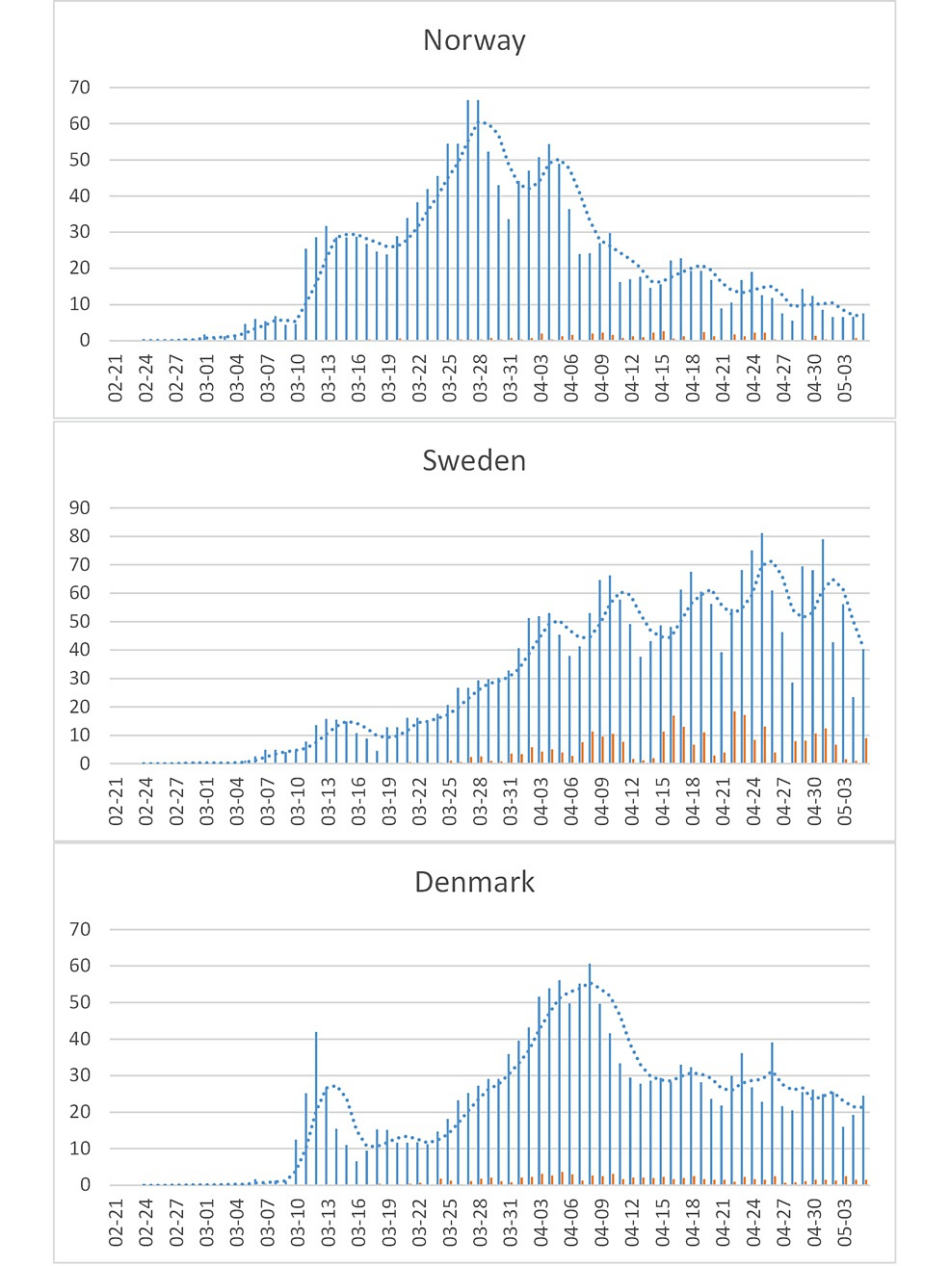
COVID-19 cases in the Scandinavia. See Figure 4 for legend.

Among the main Scandinavian countries, there was no difference in peak incidence (65/million/day, except for a recent spike in Sweden to 80) and a slower increase in incidence and a higher lethality in Sweden than in both Norway and Denmark (Figure 9).

**Figure 10:**
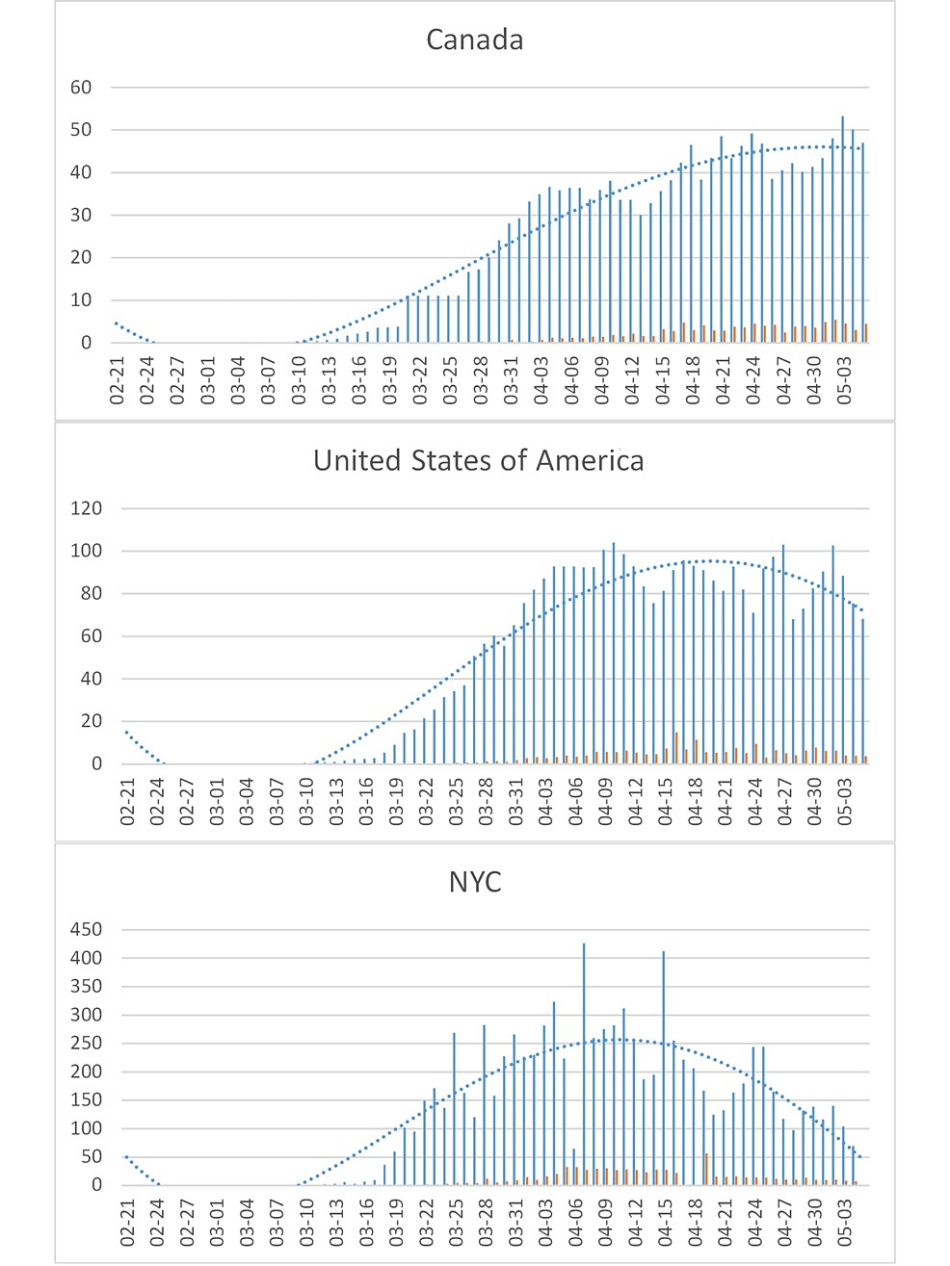
COVID-19 cases in the North America. See Figure 4 for legend. To account for an isolated peak in the Canadian data on 2020-03-26, the data for 2020-03-21-26 were averaged. NYC: New York City.

The epidemic in North America started later, especially in the USA, and most cases were imported from Europe (except for a few isolated cases likely imported directly from Asia). The increase in incidence is consistent with the dynamics of an earlier epidemic and the cumulative incidence in the USA (3,567/million), similar to Europe, as a whole (3,670/million) but still less than Spain (4,638/million). The USA reached the inflection point (half the height of the 2020-04-09/10 peak), where the rate of new cases begins to decline (when 50% of the peak incidence is reached) at about 2020-03-27, a week later than Europe’s 2020-03-21 (Figure 7). Levels in Canada remained below those in the USA and were also declining. In New York City, which is particularly hard-hit, the number of new cases seems to have peaked in early April and is also already declining. Lethality should follow suit with a delay of about a week. A particular problem arises from changes of case definitions in the midst of an epidemic, as early on in China and now in the USA (Figure 10).

Southern Hemisphere

**Figure 11:**
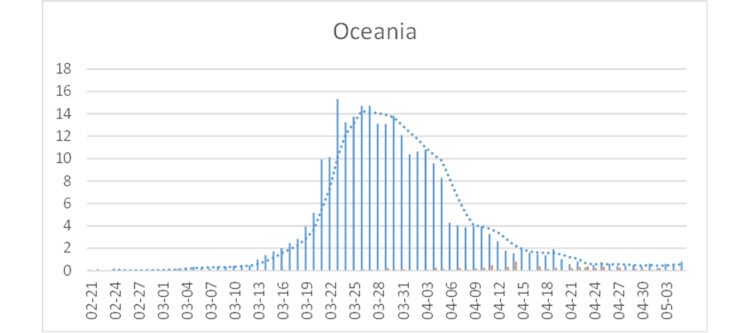
COVID-19 cases in Oceania (Australia and New Zealand). See Figure 4 for legend.

Most parts of the Southern Hemisphere (with the possible exception of Chile) have seen only few cases, but Oceania (Australia {AU} and New Zealand) shows evidence for an epidemic with a peak incidence on 2020-03-26/27. As in Canada and, earlier, in Germany and other European countries, isolated spikes tend to reflect delayed reporting, rather than changes in incident trends. The number of deaths is extremely low (Figure 11).

Modeling results for effectiveness of containment (lockdowns)

**Figure 12:**
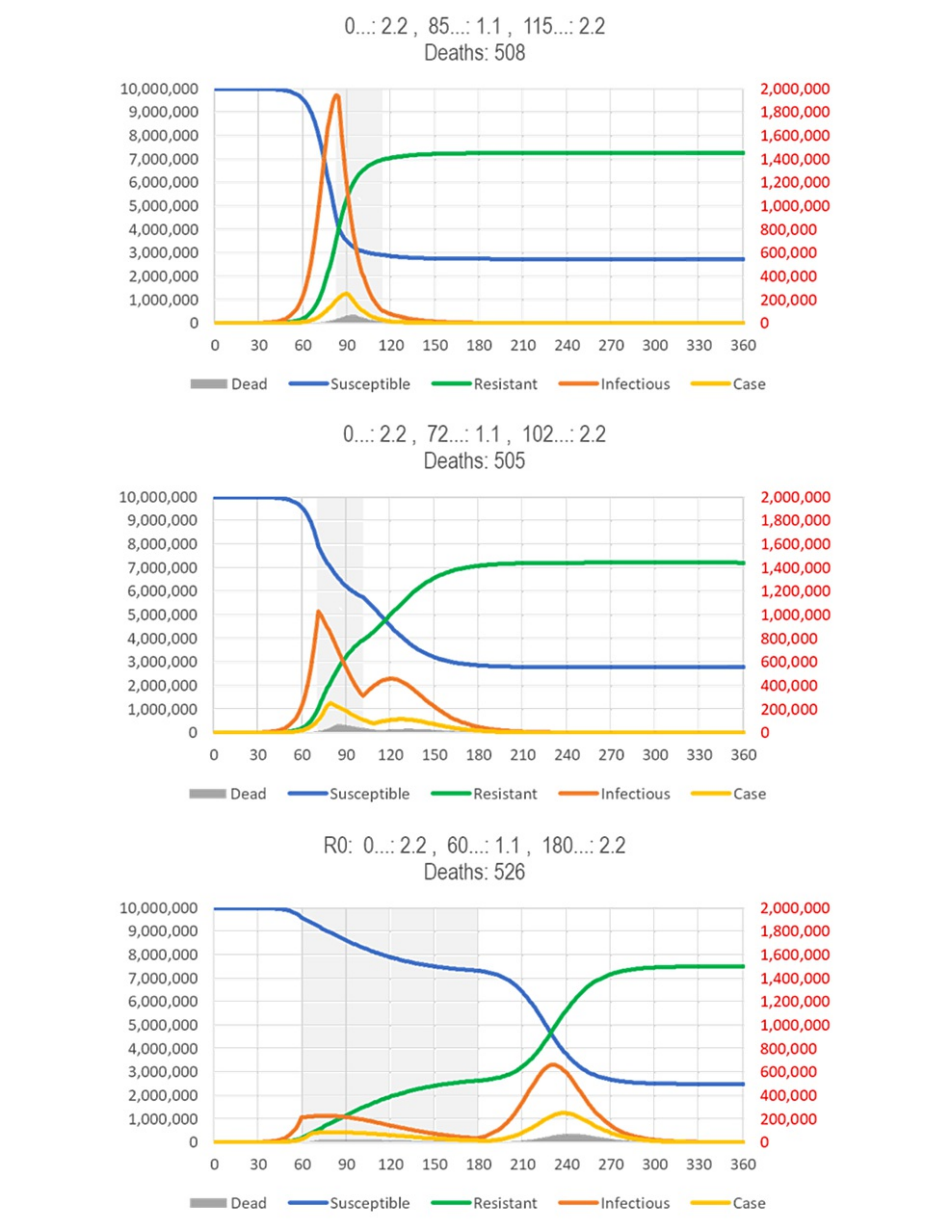
SIR model results by intervention period. Fast eradication (top), maximal reduction of deaths (middle), and early lockdown (bottom). (Top) The gray area indicates the period where containment can give a “coup de grace” to a respiratory virus disease (RVD). The narrower bell curve with a post-peak intervention indicates the reduction in the number of infections and, thus, deaths. (Middle) The gray area indicates the period where containment can have the most impact on the total number of deaths. However, the R/S ratio (duration of eradication) is smaller. (Bottom) It is assumed that a highly effective intervention reduces reproduction number (R0) by 50% for four months, beginning after the appearance of a novel type of case is noticed. The proportion of symptomatic cases (0.05%/day, i.e., 0.35% of infected people will become cases) and the proportion of cases to die (2%) may change, but the issues discussed here are broadly independent of these assumptions. See Figure 4 for legend; a spreadsheet for model calculations is available in https://app.box.com/s/pa446z1csxcvfksgi13oohjm3bjg86ql.

The effect of reducing the reproduction number by reducing the number of contacts (mitigation/lockdowns) depends on when it starts in the course of the epidemic. Figure 12 (top) shows the effect of a one-month intervention cutting R0 in half when it starts at the point of the peak prevalence of infectious subjects. Compared to the standard SIR model (Figure 3), the duration of the epidemic is shortened, albeit at the price of reducing the resistant/susceptible (R/S) ratio, so that a subsequent epidemic with the same or a similar virus (cross-immunity) could start earlier.

Figure 12 (middle) shows a one-month intervention starting about two weeks earlier, at the inflection point where the curve of the new cases changes from increasing faster to increasing more slowly. This intervention reduces the number of deaths, but the epidemic is extinguished two months later, and the R/S ratio (degree of “herd immunity” against reinfection) is lower.

**Figure 13:**
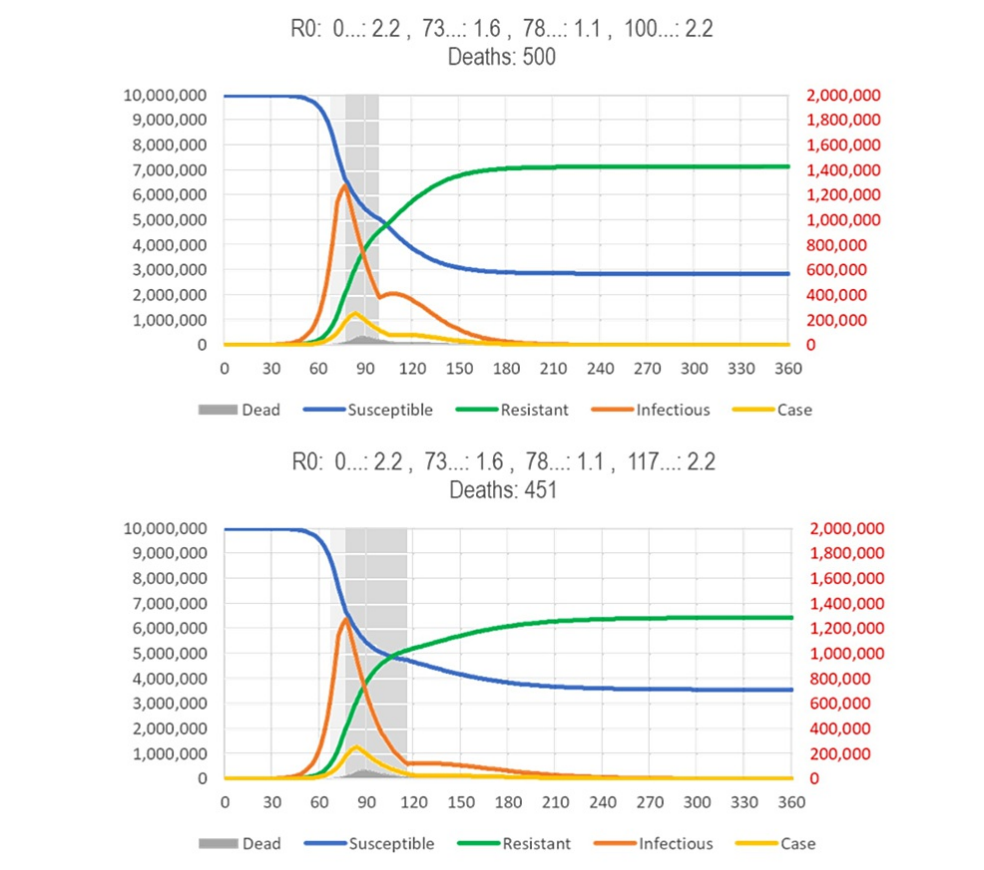
SIR model of SARS, phased in mitigation. The gray areas indicate the periods of low-intensity (five days) and high-intensity (22/40 days) restrictions (see Figure 4 for legend). SIR: susceptible-infectious-resistant; SARS: severe acute respiratory syndrome.

The SIR model is flexible enough to reflect “phased in” interventions, which start with a period of lower intensity (higher R0) to allow people to adjust, before rolling in a more restrictive intervention (higher R0). Figure 13 (top) shows an intervention starting at the same time as the intervention of Figure 12 (middle) but with a week of reduced intensity. Reducing intensity during the period where herd immunity still builds reduces the rebound and also the number of deaths. Extending the duration of the intervention (almost) extinguishes the disease (and further reduces the number of deaths by 10%).

Figure 12 (bottom) shows the detrimental effect of an intervention that starts even earlier, about two weeks before the inflection point (Figure 13, bottom). Even if the intervention is extended from one to four months, no herd immunity is created, and thus, the epidemic rebounds and will run for eight months, instead of three (no intervention) (Figure 4) or less (intervention at peak of infections) (Figure 12, top). To avoid or even reduce the rebound, one would have to end the restrictions in the lowest-risk populations (school children and young adults) first to increase the immune/susceptible ratio (the effects of targeting subpopulations differently are not accounted for in simple SIR models).

The impact of the interventions discussed on the infections (and, thus, the cases and immunity) depends mostly on the homogeneity of the population, but the number of deaths is highly dependent on how the people respond to the intervention. If parents respond to closure of schools by asking the grandparents to take care of the children, for instance, more of the elderly will get infected, and the number of deaths will increase in ways the SIR model cannot predict.

In summary, there is a narrow window of opportunity for interventions in “flattening the curve” (reducing R0) to be successful in terms of public health: Starting after the peak prevalence (of infections) has little effect (not shown). The curve goes down but is not “flattened.” Starting at the peak prevalence gives the epidemic a “coup de grace,” shortening its duration, albeit at the price of reducing the R/S ratio. The curve is narrower but also not “flattened” (Figure 12, top). Starting at the peak incidence “flattens” (and broadens) the curve and may reduce the number of deaths prevented during the current epidemic, unless (a) they increase the proportion of high-risk (elderly) people in the susceptible population at risk of becoming infected (after the children are locked away) or (b) they cause behavioral adaptations that increase the contacts of high-risk people with infectious people (such as grandparents taking care of un-schooled children while the parents are working, e.g., in hospitals), but reduces herd immunity and, thus, the chance of another epidemic coming sooner (Figure 13, bottom). To prevent a rebound, however, the intervention needs to be extended for several months (Figure 13, top). Starting before the peak incidence “flattens the curve” but also broadens it and causes a rebound, unless the intervention is continued for many more months (Figure 12, bottom).

It is herd immunity that stops the spread of an infectious disease, so in general, one would want to let the epidemic initially run its natural course (or even accelerate it, as people have traditionally done with “measles parties”) to build immunity as fast as possible. If the aim was to reduce the duration of the epidemic and its impact on the economy (and also increase the time until the next epidemic can spread), one would wait until the prevalence of infectious people reaches its peak (in the above model: day 83, red).

Without repeated broad testing, however, that peak prevalence of infections cannot be directly observed, but it is known to be followed about one week by peak number of diagnoses (new cases). This is too late to make a decision, but the SIR model shows that the peak in diagnoses is preceded by two weeks by the inflection point in cases where the curve of the new cases changes from increasing ever faster to increasing ever more slowly (day 76). The inflection point can be estimated from the observed cases in time for making a decision. (It is also about 50% of the peak number of new cases, which one might be able to predict.) Hence, peak prevalence (of infections) follows the inflection point/half peak (in number of cases) by about a week. The window of opportunity for starting an intervention is the week following the inflection point in number of diagnoses (new cases) per day.

Responses by country in relation to the inflection/decision point (as of early 2020)

Asia

China initially (on 2020-01-23) only closed means of transportation in Wuhan and other Hubei cities, causing more than 100,000 to leave the city before, in the afternoon, closing major highways. Family outdoor restrictions were issued on 2020-02-01-05 (the week following the 2020-01-31 inflection point); non-essential companies were shut down on 2020-02-13, and schools were closed on 2020-02-20, two and three weeks, respectively, after the inflection point, when interventions are not effective anymore.

South Korea controlled the epidemic without imposing a lockdown on its people and without shutting down its economy, even though the government was initially accused of complacency. People in the city of Daegu, where the Shincheonji Church is its center, were merely asked to “self-quarantine” (“voluntary lockdown”) from 2020-02-23 (the peak of the first wave). On 2020-02-29 (a day before the peak of the main wave or only three days after the inflection point), the Korea Disease Control and Prevention Agency (KCDC) issued a nationwide recommendation for “social distancing.” Still, the recommended “social distancing” may have prevented herd immunity from developing, as suggested by the continuing low number of cases becoming infected (including some deaths) (Figure 13, bottom) (see Wittkowski [1] for reference).

Europe

European governments, aiming to take advantage of being forewarned, ordered lockdowns much earlier in the epidemic. In Italy, the government ordered a nationwide close of restaurants, bars, and most stores more than a week earlier, on 2020-03-11 (Wall Street Journal {WSJ}, 2020-03-11), four days before the inflection point where the intervention prolongs the epidemic and “social distancing” may cause a rebound (Figure 12, bottom). On 2020-03-22, the lockdown in the Italian region of Lombardy was tightened to ban sports and other physical activity, as well as the use of vending machines, an intervention that could have been effective if the virus had spread in the population as a whole, but is unlikely to have changed the dynamic of the epidemic within nursing homes and, especially, to reduce the number of elderly people who died but not of the virus.

**Figure 14:**
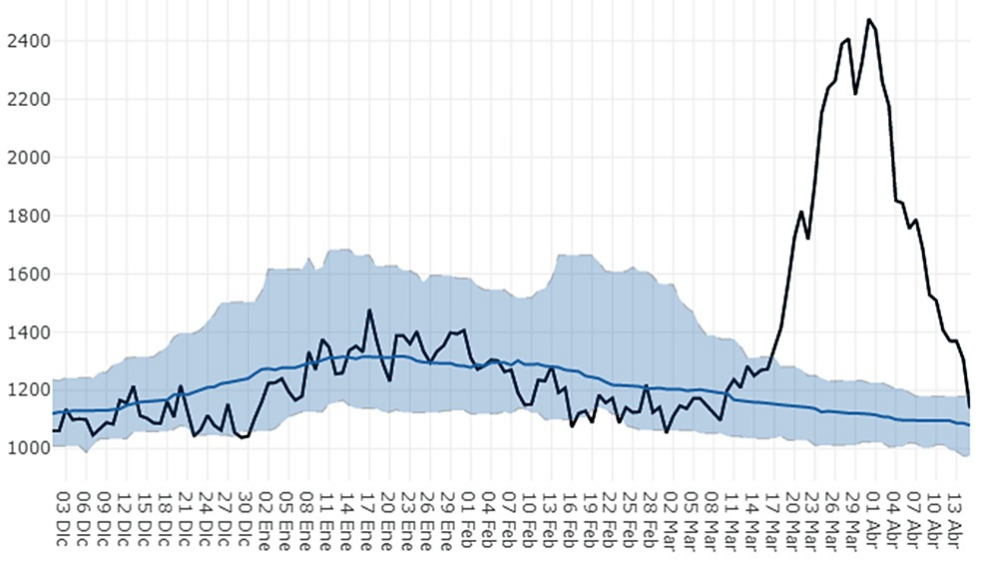
Early 2020 excess mortality in Spain. Early 2020 deaths (black line) versus estimated deaths (blue line, area: 99% confidence interval) (source defunct).

In Spain (2020), excess mortality exceeded projections for only four weeks (Figure 14), starting on the same day as the main shutdown (2020-03-14). As infections must have started and peaked about three weeks earlier, the shutdown may have hastened the decline (Figure 12, top) but couldn’t cause much “flattening” of the curve, which also has the normal, narrow, four-week width.

Germany ordered schools to be closed and various other restrictions on or before 2020-03-16/17, depending on the state and a national curfew on 2020-03-22. Taken together, “social distancing” effectively started several days before the 2020-03-22 inflection point, so the epidemic is unlikely to reach herd immunity within the near future. Hence, the epidemic will not cease for several months, unless restrictions are removed, and, thus, may rebound in autumn (Figure 12, bottom). To lessen the rebound, the Academy of Sciences Leopoldina advised the German government on 2020-04-13 to relax the shutdown because “our dignified existence in a social, cultural, and also economic way is at stake” and recommended the gradual reopening of some schools and restaurants and, in particular, to reopen primary and early secondary schools “as fast as possible.” In some states, schools were to reopen from 2020-04-20 (see Wittkowski [1] for reference).

Of particular interest is Sweden, where no lockdown was implemented. Similar peak incidence (65-80/million/day), below Europe’s 100/million/day, in all Scandinavian countries and a slower increase in incidence in Sweden than in both Norway and Denmark are consistent with the “lockdown” in the latter countries having little, if any, effect. Note that differences in lethality are characteristics of the population and, as such, also not affected by a “lockdown.” In Iceland, which also did not impose a lockdown on its citizens, the epidemic reached a peak at <40/million/day in late March and has since declined to <5/million/day.

North America

In North America, the virus arrived about a week after Europe. Responses varied not only by country but also in the USA, like in Germany, and by state. An estimated peak of 1,216-4,136 deaths per day in the USA on 2020-04-16 [9], up from the average of 1,010 on 2020-04-03/04, would be consistent with an inflection point about three weeks earlier, around 2020-03-27 (Figure 10, middle).

In NYC, one of the epicenters of the US epidemic, “social distancing” started about 2020-03-15 with closure of schools being announced and 2020-03-17 with restaurants being closed and intensified on 2020-03-22 with the closure of all non-essential businesses (similar actions were taken in New Jersey and Connecticut). Given the fast rise in number of cases in NYC turning on 2020-03-22 (Figure 10, bottom), which was also observed in Detroit, Chicago, and Dallas, an intervention at 2020-03-22 started at the inflection point (Figure 13, bottom). Hence, the virus will spread in the population much longer with “social distancing,” and a reduction of deaths through the intervention is possible (albeit not guaranteed). An inflection point of 2020-03-22 in NYC is consistent with a projected peak [9] of 524-1,090 deaths on 2020-04-10 compared to the average of 246.5 on 2020-04-02/03 (see Wittkowski [1] for reference).

In California, Executive Order N-22-20 ordered a shutdown on 2020-03-19, and many states and cities in the USA followed suit, mandating schools, restaurants, and “non-essential” businesses to close. The inflection point in the USA as a whole, however, cannot have been earlier than 2020-03-27 (Figure 10). Hence, social distancing was ordered before the epidemic reached its inflection point to “flatten” the curve. When the period the virus stays in the population is prolonged, however, incidence may rise again as soon as the intervention is ended (or ignored), causing a new “wave” until herd immunity is reached.

Oceania

Other than travel restrictions (14-day quarantine for non-residents and travel restrictions between states, similar to what other countries have enacted), the AU government has not required (although recommended) people to not go to work or closed schools or financed a bailout to prevent a burndown of the market (as in the USA). Instead, it provided funds for (see Wittkowski [1] for reference) (a) delivering an AU$20.7 billion (AU$800 per person) support package (investment, jobs, and health) and (b) opening fever clinics and funding home delivery of prescription medicines.

## Discussion

Part I: Discussion as of 2020-03

Strengths and Shortcomings

A major strength of this analysis of the epidemiological data is that it does not rely on epidemiological models with questionable assumptions. Instead, the results reflect raw incidences over time as reported by the ECDC, depicted by country or region of neighboring countries. A shortcoming of such an entirely data-based approach is that it lacks the sophistication and potential additional insights that could come from fitting, e.g., differential equation models. The only modeling assumption made is that curves should be “smooth” (except when reporting artifacts are suspected), but even then, data were redistributed only to the directly neighboring countries. Still, the evidence is strong enough to draw qualitative conclusions about possible scenarios for the spread of SARS-CoV-2 in the near future. Also, the results suggest strategies to explore the variability of the SARS-CoV-2 virus strains and to select prevention strategies.

Epidemiological Evidence for (at Least) Two Different Strains of SARS-CoV-2

During the 2003 SARS epidemic, the number of new cases peaked about three weeks after the initial increase of cases was noticed and then declined by 90% within a month. Figure 2 shows the relevant time points for the 2020 COVID-19 epidemic. The SARS-CoV-2 data also suggest that it takes at least a month from the first case entering the country (typically followed by others) for the epidemic to be detected, about three weeks for the number of cases to peak, and a month for the epidemic to “resolve.” These data are consistent with the results from the SIR model (see Epidemiological models).

The 2003 SARS and the 2020 SARS-CoV-2 are not only similar with respect to genetics (79% homology) [10], immunology [11], the involvement of endocytosis (also with influenza and syncytial viruses) [12], seasonal variation (same season in the Northern Hemisphere also with influenza, syncytial virus, and metapneumovirus) [13], and evolution (origin in bats, 88% homology) [14,15] but also with respect to the duration between emergence and peak of cases, as well as between this peak and resolution of the epidemic (Figure 2). Based on these similarities, one could have predicted the natural COVID-19 epidemic to end before 2020-04-15 in Europe and about two weeks later in the USA, but the interventions to “flatten the curve” seem to have extended the duration.

The time and height of the peak incidence of cases in the different countries are consistent with the hypothesis that SARS-CoV-2 moved step-by-step westward from China, via other Asian countries, to the Middle East (Iran, Qatar, and Bahrain), Southern Europe (Italy, followed by its neighbors CH/F/ES/AT/Slovenia {SI}), central and northern Europe, and, finally, the USA.

Viruses improve their “survival” if they develop strategies to coexist with the (human) host [16]. Multiple coronaviruses have been found to coexist in bat populations [17]. The first three months of COVID-19 data are consistent with the hypothesis that (at least) two SARS-CoV-2 strains developed. One strain, which traveled through South Korea, remained more infectious, while the other strain, which traveled through other Asian countries, lost more of its infectiousness. The strain that passed through South Korea and then Iran, Italy, and New York (SKIINY strain) showed high lethality in Iran and Italy but less lethality when it traveled to Italy’s neighbors, either because of differences in health systems, because the strain mutated back, or because a strain arriving directly from Asia had the advantage of spreading first. Only sequencing samples from these countries can help to answer these questions. Coronavirus in New York came mainly from Europe [18].

Changes in Infectivity and Lethality Between China and Europe

Mainland China is not reporting relevant numbers of new cases anymore, and Hubei reports no new cases since 2020-03-19. The number of new cases in South Korea also has declined to low levels since its peak around 2020-02-30. Maritime Southeast Asia continues to show low levels of new cases only (<3/million/day), with the possible exception of Singapore and Malaysia/Brunei. The Philippines are slowly approaching a three/million/day incidence.

The data are consistent with the same “SKII” strain traveling from China via South Korea and Iran to Italy. Iran was hit about a week after South Korea (around 2020-03-07), with a similar peak incidence but higher lethality (red, bars in Figure 1, bottom). The data also suggest a second wave of infections in Iran, with a peak at 15/million/day on 2020-03-15 and a third on 2020-04-01 with a peak of 35/million/day.

Italy was hit a week after the first wave in Iran (which peaked around 2020-03-07/08) (Figure 5, top). Incidence in Italy reached substantially higher levels than incidence reported in Iran. The peak in Italy was reached on 2020-03-22 (at about 100/million/day). Without sufficiently detailed genetic data, it is not clear whether the high lethality in Italy is due to genetic variations in the virus or to Italy having the second oldest populations in the world (after Japan). A 2020-03-20 report by the Istituto Superiore di Sanita [19], however, implicates that age and comorbidities played a role; among 3,200 deaths, mostly in Lombardy and Emilia-Romagna, median age was 80 years (interquartile range {IQR}: 73-85; only were 36 below the age of 50), and 98.8% had at least one comorbidity (hypertension: 74%, diabetes: 34%, ischemic heart disease: 30%, atrial fibrillation: 22%, and chronic renal failure: 20%).

From the epidemiological data, the virus may have spread from Italy to its neighboring countries, Switzerland, France, Spain, Austria, and Slovenia, within just a few days of arriving from Iran. The top incidence seems to be less than half of that in Italy, and the lethality is lower too. While Italy has many people 65 years and older (23%, second only to Japan), the relatively minor differences or age distribution within Europe (e.g., Germany: 21%) are unlikely to account for much of this difference. A possible explanation is that the less virulent strain(s) arriving from other parts of Asia may have had a head start in those countries so that imported infections from Italy met subjects who had already developed (cross) resistance against both strains. The parts of Europe directly hit by the SKII strain are already declining from a level of about 100/million/day; the other parts of Europe may have reached their peak of about 90/million/day (but with lower lethality) on 2020-04-04.

Predictions for COVID-19 in North America

**Figure 15:**
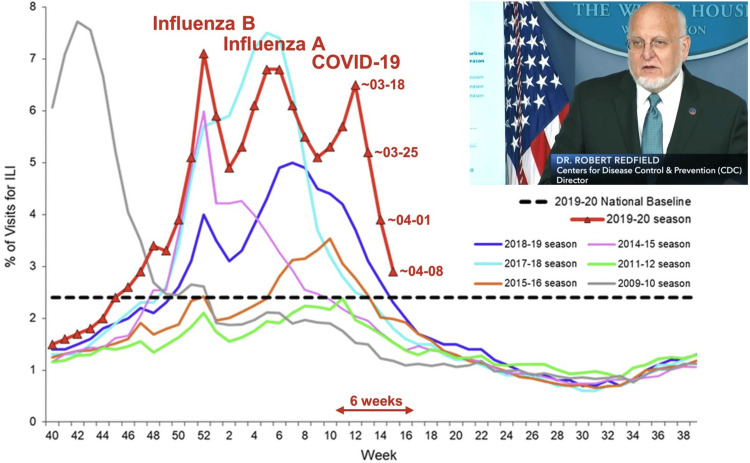
Percentage of US hospital visits of influenza-like illness (ILI). Visits were reported by the US Outpatient Influenza-like Illness Surveillance Network (ILINet). Weekly national summary, 2019/2020, and selected previous seasons were presented by the CDC director Robert R. Redfield at the 2020-04-17 White House coronavirus briefing [20]. The screenshot from Cable-Satellite Public Affairs Network (C-SPAN) is public domain.

From Figure 2, SARS-CoV-2 has arrived in the USA almost a week after it arrived in Europe, and the incidence of COVID-19 in Europe peaked around 2020-04-04. The CDC is monitoring visits to US hospitals for influenza-like illnesses (ILI). Hospital utilization (Figure 15) was highest in 2009-10 (week 42) and in early 2018-02 (week 5). In 2019/2020, the USA was hit by three epidemics: influenza in late 2019-12 (influenza B) and early 2020-02 (influenza A) and the COVID-19 epidemic where hospital visits peaked on 2020-03-16-22 (week 12), about three weeks before the peak in reported (with some delay) cases around 2020-04-09 (Figure 10) with a substantial drop by 2020-03-23-29 (week 13). By 2020-04-08, the number of hospital visits had declined for about 67% toward the national baseline.

Lethality in the USA is similar to that of Europe as a whole. Hence, there is no evidence for a highly lethal strain dominating the epidemic in the USA, with the possible exception of NYC. US incidence peaked on 2020-04-05 at about 100/million/day (34,000 cases per day). As in some European countries, the cumulative incidence could reach 4,000/million or a total of 1,300,000 cases and, at a lethality of about 3%, about 40,000 deaths. Case definitions and reporting guidelines, however, have been changed in the USA (e.g., from “death of” to “death with”) so that the number of total deaths may eventually rise to the level of about 60,000, to which the US government has lowered its predictions. A total of 28,000 deaths have already been reported. Even with this broad definition, the number of US deaths over the course of the epidemic would be within the normal range of 16,000-78,000 influenza deaths per season from pneumonia and respiratory/circulatory complications alone, which also occur predominantly among people at 65 years of age and older [21]. How many people dying will, of course, depend on their access to health care for treatment of severe complications (e.g., pneumonia or respiratory distress).

A Historical Perspective

This is not the first and likely not the last time that well-intentioned public health policies are inconsistent with our understanding of how epidemics spread. For instance, during much of the HIV epidemic, there was widespread fear that HIV could establish itself in the population as a whole, even though the data (including data showing the absence of transmission to the wives of hemophiliacs) [22] and models [23] contradicted this fear. These results have been repeatedly confirmed [24,25]. In the case of heterosexual transmission of HIV, one could argue that there was little risk associated with a the public health policy promoting condom use, but in the case of COVID-19 prevention, ignoring models and data may carry substantial risk (see Wittkowski [1] for additional reference).

During the AIDS epidemic, epidemiologists had the advantage that, in addition to the date of report, the date of diagnosis was available for analysis so that variations in reporting delays, such as mid-February in China, 2020-03-20 in Germany, and 2020-03-26 in Canada, could be accounted for. Unfortunately, the public COVID-19 data lack that information.

Implications for Prevention

In Wuhan and South Korea (and the first wave in Iran), incidence peaked after about two weeks and then declined. In Europe and the USA, it took twice as long for incidence to peak. The shorter duration of the epidemic in China and South Korea, however, does not demonstrate the effectiveness of social distancing because the social distancing started too late to be effective. The longer duration in Europe is consistent with premature interventions (aiming to “flatten the curve”) prolonging the epidemic (“flattening” = “broadening”).

A major problem with respiratory diseases is that one cannot stop all chains of infections within families, friends, and neighbors. Even after a couple of weeks of “lockdown,” there will be a few infectious persons, and as long as there are enough susceptible people in the society, this is enough to re-start the epidemic until there are enough immune people in the society to create “herd immunity.” Hence, one would expect the cases to appear in waves (Figure 13) (the period of the “lockdown” corresponds to March to May (2020) in the USA). Such waves of cases have been seen in different countries, and the longer than expected duration of the epidemic supports the hypothesis that the social distancing/lockdown interventions had some effect, albeit at a high cost for ~10% of deaths saved.

The epidemic of Oceania, together with those of China, South Korea, and Iran, is consistent with the model results suggesting that a natural COVID-19 epidemic peaks two weeks after the first cases are seen and then declines with financial and medical assistance from the government to prevent deaths to reduce the burden on the health system and damage to the economy.

The data from Scandinavia (Figure 9) also do not support the effectiveness of social distancing. The epidemic curves of Sweden and the surrounding Norway, Denmark, and Finland do not indicate that imposed “social distancing” rules in the latter countries, but not in Sweden, had a major impact. Iceland, which, like Sweden, did also not impose a lockdown on its citizens, had a particularly mild epidemic.

This analysis of the publicly available data suggests that at the time Italy imposed quarantine on the Lombardy and adjacent regions on 2020-03-08, the SKII virus strain had already reached the adjacent countries (Switzerland, France, Spain, Austria, and Slovenia), even though the lockdown started early (2020-03-08), which may have caused a rebound when compliance declines.

Some containment strategies could even be counterproductive in other ways. For instance, the simple SIR model does not account for age stratification. In diseases such as COVID-19, children develop mostly mild forms; elderly people have a high risk of dying [26]. Hence, containment of high-risk groups, such as elderly people in nursing homes (see the Washington State example), is highly effective in protecting them from becoming infected to reach herd immunity. A substantial increase in the duration of the epidemic by preventing immunity to develop among the young, however, might make effective containment of the elderly more difficult and, thus, increase the number of deaths among the elderly.

Part II: Discussion added more than two years later

Having been published initially on 2020-03-31, only a week after lockdowns started in the USA (with updates here until 2020-05-05), several shortcomings need to be acknowledged. First, the initial predictions were based on South Korea as the first country with a complete first wave by that time outside of China, and at least during the first year of the epidemic, South Korea fared much better than many other countries. Second, escape strains of RVDs had not been seen since 1919, and the first evidence for virus escape did not emerge until 2020-10 [27]. With the frequent emergence of strains resistant to natural and vaccine-induced immunity against previous strains, “broad-spectrum antiviral interventions” [12] are urgently needed, but pharmacological interventions have not been as successful as hoped for. Since the “pre-existing pathologies” identified in early 2020-03 in Italy [19] (including cardiovascular, diabetes, and obesity) are still major risk factors for death from COVID-19, behavioral (“non-pharmacological” [2]) interventions, including intermittent fasting (mimetics), should have been mentioned [28]. Also, as discussed above, the simple SIR model does not account for population stratification, and thus, the higher risk of the vulnerable population from universal mitigation could not be accounted for. Finally, the “SKII” variant predicted from the epidemiological data was identified from sequencing data as the D614G mutation in 2020-07 [29] and confirmed in 2020-09 [30].

## Conclusions

Part I: Conclusions as of 2020-04

Until a vaccine becomes available, the only pharmacological strategy to reduce the number of deaths is to reduce the damage the infection (and immune system) does, e.g., by reducing the initial viral load and making sure that people get treated at the earliest signs of pneumonia. Aside from separating susceptible populations (elderly and high-risk subjects, e.g., in nursing homes; see the first US case diagnosed on 2020-02-28 in Seattle, WA) from the epidemic, which is effective as long as the virus is circulating, public health intervention aiming to contain a respiratory disease needs to start within a narrow window of opportunity, i.e., when or a week after the curve of the new cases changes from increasing faster to increasing more slowly. Then, the epidemic must generate a sufficient number of immune people, before containment efforts can stop, which is typically after about a month or two (depending on whether containment started late or early, respectively). When the window of opportunity has been missed, any type of containment has only a limited impact on the course of the epidemic but a high price to pay for the economy and society.

So far, the hospital systems in most affected countries have shown to be able to handle the COVID-19 epidemic. As epidemics in maritime Asia and the Southern Hemisphere remain a possibility and some may develop within the next weeks, the use of scientifically sound models may help to time interventions to optimize their effect. In particular, the risk of prolonging the epidemic and increasing the number of deaths rises when interventions (“flattening the curve”) start before the inflection point in the number of cases is reached.

To determine that time point, case data collected and reported need to contain not only the date of report but also the date of “diagnosis” and whether the patient had clinical symptoms or was merely tested positive and whether the patient was positive for circulating virus RNA/DNA (currently infectious) or merely for antibodies (already immune).

Part II: Epilogue (added more than two years later)

The main results published in early 2020 stood the test of time. The conclusion that lockdowns would not routinely end an RVD epidemic has proven correct time and again. Hence, well-established, fully disclosed, and broadly discussed epidemiological models such as the SIR model used here can provide useful insights into the dynamics of epidemics and, thus, could help with working toward a consensus among epidemiologists, sociologists, physicians, and virologists emerging rather than dictated through censorship. Such an open discussion could improve the risk/benefit ratio of political decisions about how to respond to the tidal wave of an epidemic, especially when signs of the virus evolving (here: the SKII/D614G variant) are seen early on in the epidemic.
